# Camurati-Engelmann Disease Association With Hypogonadism and Primary Hypothyroidism

**DOI:** 10.5812/ircmj.9481

**Published:** 2014-08-01

**Authors:** Soo Fin Low, Norzailin Abu Bakar, Chai Soon Ngiu

**Affiliations:** 1 Department of Radiology, University Kebangsaan Malaysia Medical Centre, Kuala Lumpur, Malaysia; 2 Department of Medicine, University Kebangsaan Malaysia Medical Centre, Kuala Lumpur, Malaysia

**Keywords:** Camurati-Engelmann Syndrome, Hyperostosis, Primary Hypothyroidism

## Abstract

**Introduction:**

Camurati-Engelmann disease (CED) is a rare autosomal dominant disease with various phenotypic expressions. The symptoms usually develop during childhood. The hallmark of the disease is bilateral symmetric diaphyseal hyperostosis of the long bones with progressive involvement of the metaphysis. The epiphysis is strictly spared. The common clinical symptoms are pain of the extremities, muscle wasting, waddling gait, and lethargy. CED is rarely seen in conjunction with hypogonadism. CED-associated hypothyroidism has not been reported yet. Clinical assessment and skeletal survey are important to make the diagnosis.

**Case Presentation:**

Hereby we reported a case of CED with concomitant hypogonadism and hypothyroidism. Serial plain radiographs of the patient showed classic and progressive diaphyseal cortical hyperostosis of the long bone.

**Conclusions:**

Hyperostosis of the skull was observed in the present case. The characteristic osseous changes of CED were highlighted and the differential diagnoses were discussed.

## 1. Introduction

Camurati-Engelmann disease (CED), also known as progressive diaphyseal dysplasia, is a form of craniotubular hyperostosis. It is a rare autosomal dominant disease with various genotypes and phenotypes. Mutation of the transforming growth factor β1 (*TGFβ1*) gene in chromosome 19q13 is the leading cause of CED (1). CED was first reported by Cockayne in 1920 (2). Two years later, Camurati suggested its hereditary nature (3) and Engelmann reported a case of muscular wasting and marked bone dysplasia in 1929 (4). The term “progressive diaphyseal dysplasia” signifies the progressive hyperostosis with invariable involvement of the diaphysis (5); however, “Camurati-Engelmann disease” has become the widely used eponym.

## 2. Case Presentation

The presented case was a 31-year-old Chinese woman who was diagnosed with CED at the age of seven. Her antenatal and neonatal histories were uneventful. She had five siblings and she was the fourth child of her parents. Her eldest brother, who was 38 years old, developed CED symptoms at three years of age and the diagnosis was established six months following the initial presentation. Her parents and other siblings were asymptomatic; however, no investigation had been performed on them. 

Although her symptoms started at one year of age, due to financial constrained and poor social support, her parents brought her to seek medical treatment at the age of seven. At first, she presented with generalized muscle wasting of all four limbs, more prominently in lower extremities. Subsequently, she experienced progressive worsening of the bone pain. She had poor appetite and failure to thrive. At the age of seven, she was brought to the hospital due to unbearable lower limb pain. Physical examination revealed severe muscle and subcutaneous fat wasting. Her body weight was below fifth percentile. She walked with waddling gait. Femur radiograph was obtained and showed irregular sclerosis of the bone. Unfortunately, the image could not be obtained for review. Before the family history was disclosed and based on the clinical features and her femur radiograph, the initial diagnosis was muscular dystrophy with chronic osteomyelitis. Later, she was reviewed in pediatric clinic and her family history was obtained. Finally, the diagnosis of CED was made based on the clinical features, radiographic findings, and family history.

**Figure 1.**
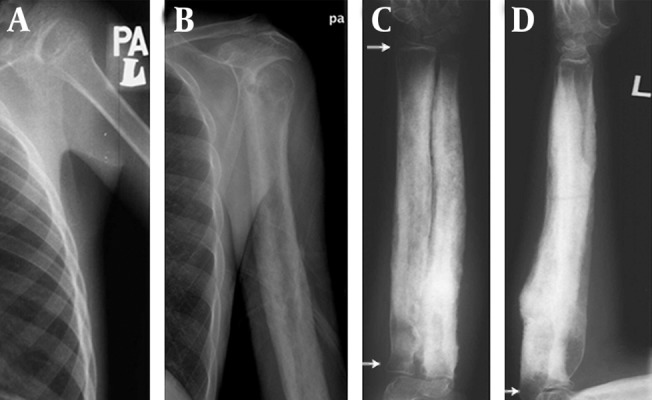
Skeletal Survey A, normal appearance the proximal meta-diaphysis of the humerus had at 15 years of age. B, typical cortical hyperostosis of the humerus meta-diaphysis with sparing of the epiphysis at 28 years of age. C and D, left radioulnar radiograph at the age of 24 years shows meta-diaphyseal hyperostosis of the ulnar and radius with sparing the epiphysis (arrows).

**Figure 2.**
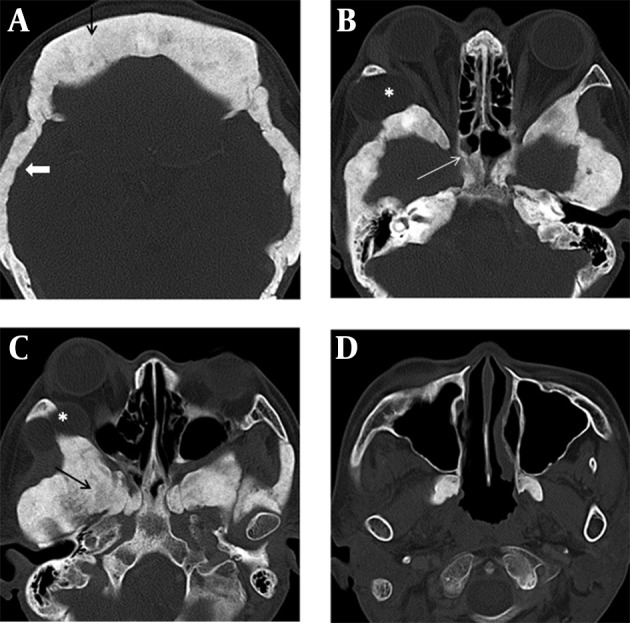
The Maxillary Sinus A, computed tomography of orbit shows hyperostosis of the skull base with complete obliteration of the dipolic space (thick arrow) and frontal sinus (thin arrow). B, the walls of the sphenoid sinuses are thickened and sclerotic (white arrow). C, skull base foramina are encroached (black arrow). D, the maxillary sinus and mandible are spared. B and C, a dermoid cyst at the lateral angle of the right orbit (asterisk).

At the age of 20, she was also diagnosed with concomitant hypogonadism and primary hypothyroidism. Her thyroid gland had not been enlarged but her free T4 level was low and thyroid stimulating hormone level was high. The symptoms of hypothyroidism resolved with initiation of L-thyroxine. Her hearing was not compromised. During follow-up, serial chest radiographs were obtained and showed typical progressive diaphyseal hyperostosis of the humerus (Figure 1, A and B). The rib, clavicle, and vertebrae were spared. Her left radioulnar radiograph that was taken at the age of 24 years also showed similar changes (Figure 1, C and D). A computed tomography of orbit was performed recently due to progressive right exophthalmos. A lateral periorbital dermoid cyst was found but she was not eager for surgical removal. Moreover, the computed tomography showed classic skull changes of CED, which included extensive sclerosis and thickening of the frontal, temporal, and petrous bones, resulting in obliteration of diploe spaces and encroachment into the frontal and sphenoid sinuses. The maxillary sinus was relatively spared (Figure 2). Her orthopantomogram (OPG) showed no hyperostosis of the mandible.

Similarly, her eldest brother had concomitant hypogonadism and primary hypothyroidism. His clinical presentation was similar to the patient but had manifested in more severe form. He was wheelchair-bound since the symptoms started due to severe muscle wasting and bone pain. He had bilateral mixed conductive and neurosensory hearing impairment. His physical examination showed mild hepatosplenomegaly.

The results of their full blood counts, serum urea, electrolytes, calcium, and liver function test were in normal range, except for persistently raised alkaline phosphates (180-265 U/L). They required long-term analgesia to relieve pain. Due to the disease progression and chronic bone pain, they were not able to adapt to normal social parameters. Both of them were unemployed and required social welfare support.

## 3. Discussion

CED is a rare autosomal dominant disease due to *TGFβ1* gene mutation. The disease symptoms usually develop during childhood and before the age of 30 (1). The process of intramembranous osseous formation and remodeling is disturbed (1). However, CED has variable penetrance, even within the families, with genetic anticipation in successive generation (6). It is characterized by bilateral symmetric hyperostosis of the diaphysis of the long bones, which cause bony expansion and progressive obliteration of the medullary cavity. The tibia and femur are affected first (1). As the disease progresses, the changes extend to the metaphyses but spare the epiphyses as what was observed in the present case. Patients with CED commonly presented with bone pain (68%), waddling gait (48%) and fatigue (44%), and muscle weakness (39%). Significant percentage of the patients show wasting of the subcutaneous fat (21%) and muscles (39%), which is attributed to the mutation of *TGFβ1* gene (1).

In the present case, examination of hearing and visual acuity revealed normal findings, albeit hyperostosis of the skull base led to obliteration of the foramina. Hyperostosis of the skull base tends to encroach into the foramina and may lead to sensory deficits, blindness, or hearing loss due to nerve or blood vessels compression (7). It was reported that 54% of the patients with CED had skull base involvement (1). Carlson et al. evaluated 306 patients with CED and reported that among 173 patients (56.5%) with skull base hyperostosis, less than one-fourth of them were symptomatic (7).

Systemic manifestation such as anemias, leucopenia, and hepatosplenomegaly are seen occasionally (8). Although biochemistry findings are usually normal in CED, abnormal values for several markers of bone formation and resorption have been reported (9). Minority of the patients have raised alkaline phosphatase, as in the present case. A paper postulated that hypogonadism in CED could be attributed to gonadal growth and steroidogenesis regulation impairment, in which *TGFβ1* has an important role (10). CED association with hypothyroidism has not been reported yet. *TGFβ1* plays multiple roles in controlling proliferation, differentiation, and apoptosis of many cell types. The effect of mutated *TGFβ1* on thyroid gland is not known. We believed that the presence of hypothyroidism in this patient and her brother was most likely related to *TGFβ1* mutation rather than just coincident. *TGFβ1* mutation might have a direct effect on the thyroid gland or might affect the pathway of thyroid hormone synthesis.

Hyperostosis of the bone is well depicted on plain radiograph. Computed tomography has the advantage in demonstrating the skull base foramina encroachment. Scintigraphy allows early recognition of the disease by showing increased osteoblastic activity of the bones before sclerosis become notable on plain radiograph (1).

There are several conditions that mimic the radiographic changes of CED. The differential diagnoses include ribbing disease (multiple diaphyseal sclerosis), chronic osteomyelitis, familial hyperphosphatasia, and Paget’s disease. Ribbing disease is a rare autosomal recessive bony dysplasia with clinical manifestations similar to CED. Nonetheless, ribbing disease presents in adults with unilateral or asymmetric bilateral long bone involvement (11). No skull involvement, neurological deficit, abnormal gait, or anemia is seen in ribbing disease. Histopathologic examination is the most reliable method to differentiate these two entities. In ribbing disease, only osteoblastic activity is affected and Haversian system is obliterated. In CED, both osteoblastic and osteoclastic activities are affected. The Haversian system is either normal or enlarged (12).

Chronic osteomyelitis usually affects a single bone but CED shows multiple and bilateral symmetric long bones involvement. Osteomyelitis most commonly involves metaphysis but CED affects the diaphysis of long bones. The onset of familial hyperphosphatasia is at about 18 month of birth. The clinical features are painful bowing limbs, pathological fracture, muscle weakness, abnormal gait, and rapid enlargement of the calvarium. Symmetric cortical thickening of the long bones with sparing of the epiphysis are identical in both familial hyperphosphatasia and CED; however, short bones of the hands and feet are only affected in familial hyperphosphatasia. Moreover, no bowing of the long bone is seen in CED. In addition, CED rarely presents with low-impact pathologic fracture (13).

Cortical thickening of the skull bone and radiologic feature of CED are also present in Paget’s disease; however, it is unusual to diagnose Paget’s disease before the age of 40. Paget disease causes expansion of the diploic space but CED obliterates the diploic space. Malignant transformation is possible in Paget’s disease but it has been never reported in CED. Steroid and analgesia are used to relieve bone pain in CED. Individual on bisphosphonate may show different response to treatment as biphosphonates may improve or aggravate their symptoms.

In conclusion, diagnosis may not be possible by radiographic findings alone. Clinical information is essential in establishing the diagnosis. Mutation in *TGFβ1* gene disturbs the intramembranous bones, apoptosis of the fat and muscle, the gonadal growth, and steroidogenesis regulation. Mutation in *TGFβ1* gene may affect the thyroid gland or the pathway of thyroid hormone synthesis.
